# Polyphosphate Kinase 2: A Novel Determinant of Stress Responses and Pathogenesis in *Campylobacter jejuni*

**DOI:** 10.1371/journal.pone.0012142

**Published:** 2010-08-17

**Authors:** Dharanesh Gangaiah, Zhe Liu, Jesús Arcos, Issmat I. Kassem, Yasser Sanad, Jordi B. Torrelles, Gireesh Rajashekara

**Affiliations:** 1 Food Animal Health Research Program, Ohio Agricultural Research Development Center, Department of Veterinary Preventive Medicine, The Ohio State University, Wooster, Ohio, United States of America; 2 Center for Microbial Interface Biology, The Ohio State University, Columbus, Ohio, United States of America; Charité-Universitätsmedizin Berlin, Germany

## Abstract

**Background:**

Inorganic polyphosphate (poly P) plays an important role in stress tolerance and virulence in many bacteria. PPK1 is the principal enzyme involved in poly P synthesis, while PPK2 uses poly P to generate GTP, a signaling molecule that serves as an alternative energy source and a precursor for various physiological processes. *Campylobacter jejuni*, an important cause of foodborne gastroenteritis in humans, possesses homologs of both *ppk1* and *ppk2*. *ppk1* has been previously shown to impact the pathobiology of *C. jejuni*.

**Methodology/Principal Findings:**

Here, we demonstrate for the first time that the deletion of *ppk2* in *C. jejuni* resulted in a significant decrease in poly P-dependent GTP synthesis, while displaying an increased intracellular ATP:GTP ratio. The Δ*ppk2* mutant exhibited a significant survival defect under osmotic, nutrient, aerobic, and antimicrobial stresses and displayed an enhanced ability to form static biofilms. However, the Δ*ppk2* mutant was not defective in poly P and ppGpp synthesis suggesting that PPK2-mediated stress tolerance is not ppGpp-mediated. Importantly, the Δ*ppk2* mutant was significantly attenuated in invasion and intracellular survival within human intestinal epithelial cells as well as in chicken colonization.

**Conclusions/Significance:**

Taken together, we have highlighted the role of PPK2 as a novel pathogenicity determinant that is critical for *C. jejuni* survival, adaptation, and persistence in the host environments. PPK2 is absent in humans and animals; therefore, can serve as a novel target for therapeutic intervention of *C. jejuni* infections.

## Introduction

*Campylobacter jejuni*, a Gram-negative microaerophilic bacterium, frequently causes gastroenteritis in humans and accounts for up to 15% of all diarrheal cases worldwide [1], [2]. Human infections with *C. jejuni* are characterized by a rapid onset of fever, diarrhea, abdominal pain and vomiting. Although self-limiting in the majority of the population, *C. jejuni* infections are also associated with Guillain-Barré Syndrome [3], Reiter's syndrome [4], inflammatory bowel syndrome [5] and immunoproliferative small intestinal disease [6]. *C. jejuni* is a zoonotic pathogen that exists as a commensal in the gastrointestinal tract of chickens and mammals [7], [8]. Human infections are primarily acquired through consumption of contaminated chicken and other livestock meat, contaminated water, and unpasteurized milk [1]. Despite its public health significance, relatively little is known about the molecular mechanisms contributing to *C. jejuni* stress tolerance, host colonization, and pathogenesis.

Inorganic polyphosphate (poly P), a phosphate polymer, plays an important role in bacterial survival, stress tolerance and virulence in many bacterial species [9]. This is not surprising since poly P is involved in several housekeeping functions such as reservoir for phosphate and energy, chelator of metals, component of membrane channel for DNA entry, component of bacterial capsule, and buffer against alkali [10]. Additionally, poly P is essential in several pathogenic bacteria for stress and virulence-related functions [9], [11], [12]. Several specialized enzymes are involved in poly P metabolism. Polyphosphate kinase 1 (PPK1) is responsible for reversible synthesis of the majority of poly P in the cell [13], [14]. The *ppk1* deletion mutants in many bacterial pathogens show diverse phenotypes including defects in stress responses, motility and virulence [9], [14], [15], [16], [17], [18], [19], [20], [21]. Many bacterial species contain another enzyme, PPK2, which preferentially mediates poly P-driven generation of GTP [22], [23], a molecule known to have important roles in cell signaling as well as DNA, RNA, protein, and polysaccharide synthesis [24], [25]. In addition, PPK2 is an important virulence factor as it regulates intracellular survival in *Mycobacterium* [23]. PPK2 is widely conserved in bacteria including major human pathogens such as *Mycobacterium tuberculosis*, *Pseudomonas aeruginosa* and *Vibrio cholera* [22]. However, with the exception of a recent report on PPK2 from *Mycobacteria* [23], previous studies have focused on only characterizing the structure and enzymatic activity of PPK2 [22], [26], [27] and little effort has been made towards understanding the role of PPK2 in stress responses and pathogenesis.

The genome of *C. jejuni* possesses homologs of both *ppk1* and *ppk2* genes. Previously, we and others have demonstrated the role of PPK1 in *C. jejuni* stress survival, adaptation and *in vivo* colonization [15], [16]. However, to our knowledge, there have been no studies addressing the role of PPK2 in *C. jejuni* physiology, stress tolerance and *in vivo* pathogenesis. Here, we examined the role of PPK2 in *C. jejuni* patho-physiology by generating a *ppk2* mutant in a highly virulent strain of *C. jejuni*. We report that the *Δppk2* mutant displayed deficiency in several survival- and virulence-related phenotypes, which emphasizes a role for PPK2 in mediating stress tolerance and pathogenesis in *C. jejuni*.

## Results

### The *Δppk2* mutant is impaired in GTP synthesis and exhibits increased intracellular ATP:GTP ratio

**Figure 1:**
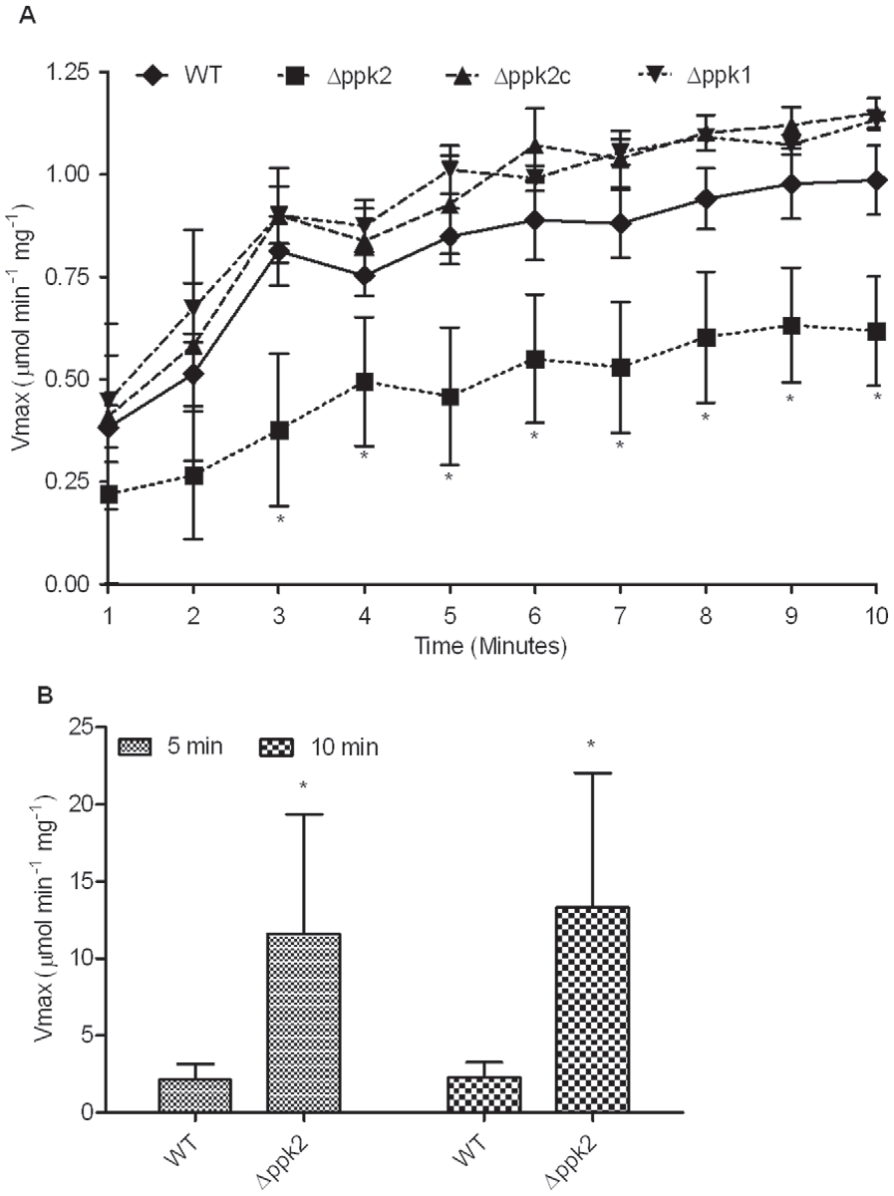
Poly P-dependent GTP and ATP synthesis in the *Δppk2* mutant. (**A**) The *Δppk2* mutant is defective in poly P-dependent GTP synthesis. GTP synthetic activity was determined spectrophotometrically using a modified enzyme coupled assay. (**B**) The *Δppk2* mutant exhibits increased intracellular ATP:GTP ratio. Poly P-dependent ATP synthesis was determined spectrophotometrically using a modified enzyme coupled assay. ATP synthesis was measured for up to 10 min. Only data for 5 and 10 min are shown. Both assays were repeated 3 times with 3 replicates in each assay and the data was expressed as mean±SE. * *P*≤0.05.

PPK2 from *P. aeruginosa* and *Mycobacterium* species mediate preferential generation of GTP using poly P as a phosphate donor [22], [23]. Therefore, we assessed the ability of *C. jejuni Δppk2* mutant to generate GTP from poly P. The *C. jejuni Δppk2* mutant was significantly (*P*≤0.05) defective in poly P-dependent GTP generation compared to WT (Figure 1A), while complementation restored the ability of the *Δppk2* mutant to generate GTP to levels comparable to WT (Figure 1A). Further, consistent with the enzyme assay, thin layer chromatography (TLC) analysis of the reaction mixture revealed 1.2 fold lower GTP level in the *Δppk2* mutant compared to WT. The *C. jejuni Δppk2* mutant, however, showed no general growth defect in rich medium (Figure S1). This observation led us to hypothesize that the *C. jejuni Δppk2* mutant possesses an alternative source for GTP required to support normal growth. *P. aeruginosa* possesses the alternative NTP-generating enzymes, nucleoside diphosphate kinase (NDK) and pyruvate kinase (PK) [25]. PK generates NTPs using phosphoenolpyruvate as a phosphate donor, while NDK synthesizes NTPs using ATP as a phosphate donor. PK is sensitive to Tween 20, while NDK is not. Therefore, we monitored the growth of the Δ*ppk2* mutant in MH broth containing 0.1% (v/v) Tween 20. The presence of Tween 20 did not affect the growth of the *C. jejuni Δppk2* mutant in rich medium (data not shown).

Since PPK2 can mediate poly P-driven ATP synthesis in other bacteria [27], we investigated whether *C. jejuni* PPK2 has a role in ATP synthesis. Interestingly, the *Δppk2* mutant displayed a significant increase (*P*≤0.05) in the ability to generate poly P-dependent ATP compared to WT (Figure 1B). Consistent with our enzyme assay, densitometry analysis measuring ‘*de novo*’ ATP production by TLC showed that the *Δppk2* mutant had 1.6 fold higher ATP levels compared to WT.

### The ***Δ*** *ppk2* mutant and parental strain exhibit similar ppGpp and poly P levels

**Figure 2:**
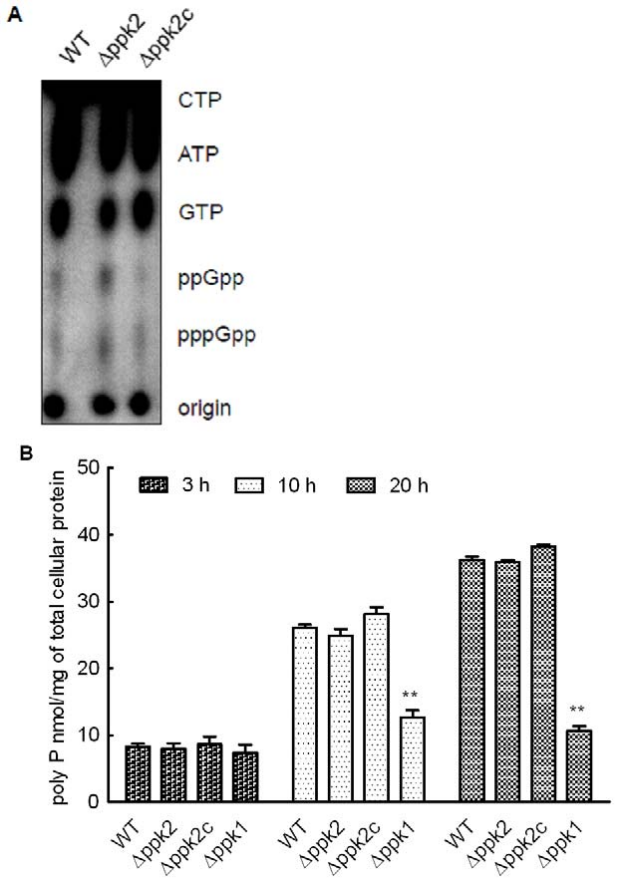
ppGpp and poly P levels in the *Δppk2* mutant. (**A**) The *Δppk2* mutant shows similar ppGpp levels to that of WT under nutrient downshift. The amount of ppGpp accumulation was assessed by growing the bacterial strains in MH broth microaerobically to early log phase (OD_600_ of 0.25) and labeling in nutrient poor medium (MOPS-MGS) with ^32^P. Nucleotides were resolved by TLC and visualized using autoradiography. (**B**) Poly P accumulation in the WT and *Δppk2* mutant at different growth phases. The amount of poly P in the cell was determined by toluidine blue O method. Each data point is the mean±SE of 3 independent experiments. ** *P*≤0.01.

Since GTP serves as the precursor for ppGpp synthesis [28], we asked whether PPK2 has a role in ppGpp accumulation. Surprisingly, though the *Δppk2* mutant displayed slightly elevated ppGpp accumulation compared to WT (Figure 2A), there was no significant difference in the ppGpp accumulation either after 1 hr or 3 hr labeling. Additionally, densitometry analysis also revealed no statistically significant difference in the amount of ppGpp between the WT and *Δppk2* mutant.

Though GTP synthesis is the preferred function, PPK2 is also involved in poly P synthesis in other bacteria [26]. Therefore, we asked whether PPK2 has a role in poly P accumulation in *C. jejuni*. The *Δppk2* mutant exhibited similar poly P levels to that of WT (Figure 2B). However, as previously reported [15], [16], the *ppk1* mutant showed a significant (*P*≤0.01) defect in poly P accumulation (Figure 2B) suggesting that *ppk1* but not *ppk2* is involved in poly P synthesis in *C. jejuni*.

### PPK2 is essential for *C. jejuni* nutrient and osmotic stress survival as well as aerotolerance

**Figure 3:**
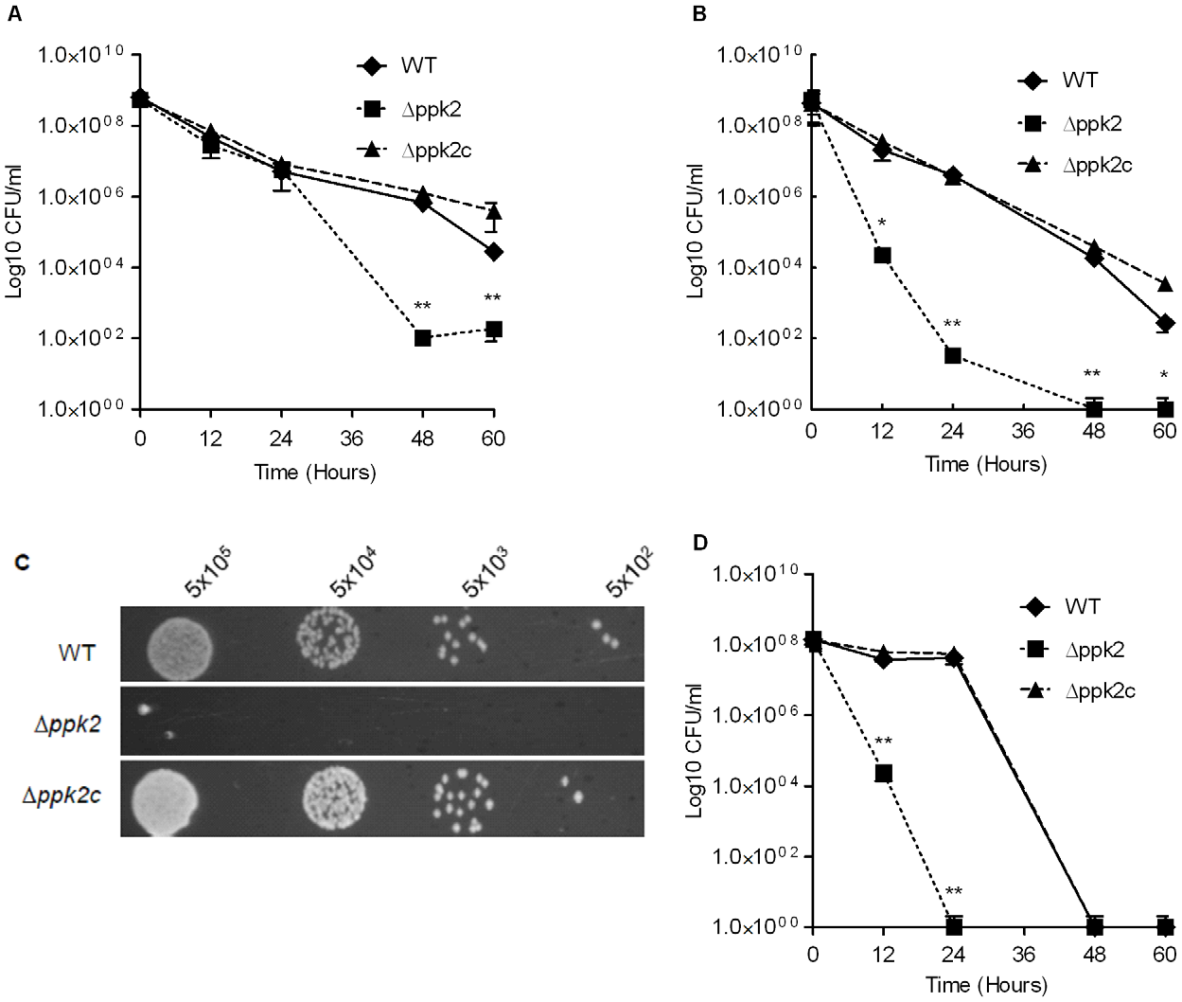
Survival of the *Δppk2* mutant under nutrient, osmotic and aerobic stresses. (**A**) Survival of *C. jejuni* under nutrient downshift. Survival under nutrient stress was assessed by growing bacterial strains in MEM and determining the CFU at different time points. (**B**) Survival of *C. jejuni* under osmotic stress. Survival under osmotic stress in liquid culture was assessed in MH broth containing 0.25 M NaCl. (**C**) Survival of *C. jejuni* under osmotic stress in solid medium. Survival in solid medium was determined by spotting 5×10^2^–5×10^5^ CFU onto MH agar containing 0.17 M NaCl. (**D**) Survival of *C. jejuni* under aerobic stress. Survival of the *C. jejuni* 81–176 WT and *Δppk2* mutant was assessed by determining the CFU of aerobically grown bacteria at different time points. For all assays, each data point represents the mean±SE of 3 independent experiments. * *P*≤0.05 and ** *P*≤0.01.

To assess the contribution of PPK2 to *C. jejuni* survival under nutrient stress, we monitored the survival of the *Δppk2* mutant in MEM. The *Δppk2* mutant was significantly (*P*≤0.01) impaired in survival in MEM compared to WT only during stationary-phase (Figure 3A). After exposure to nutrient stress, the *Δppk2* mutant had 3.5-log and 2.1-log fewer bacteria than the WT strain at 48 and 60 h, respectively, while complementation of the *Δppk2* mutant restored the survival defect to levels comparable to WT (Figure 3A). This finding is in contrast to the *ppk1* mutant [15], [16] where a nutrient survival defect was evident at both log and stationary-phases of growth. For *P. aeruginosa,* it is shown that PPK2 is induced >100-fold when the culture approaches stationary phase as a consequence of the GTP requirement for different cellular processes during this growth stage [9]. Thus, the defect in GTP synthesis observed during stationary phase in the nutritionally limited media may explain the reduced ability of the *Δppk2* mutant to survive in the stationary phase.

*C. jejuni Δppk2* mutant was significantly (*P*≤0.01 or 0.05) defective in survival under osmotic stress both in liquid culture and on solid medium compared to WT strain (Figure 3B and 3C). After 0.25 M NaCl treatment in liquid culture, the *Δppk2* mutant exhibited a 3- and 5-log reduction in bacterial numbers compared with WT at 12 and 24 h, respectively. While at 48 and 60 h, no mutant bacteria were recovered compared to WT which showed 1.8×10^4^ and 2.7×10^2^ CFU of bacteria, respectively. Similarly, the *Δppk2* mutant failed to grow on solid medium in the presence of 0.17 M NaCl. The *Δppk2* mutant grew only when 5×10^5^ CFU were spotted, while the WT and complemented strains grew even when 1000 fold less CFU were spotted (Figure 3C, a difference of 1000-fold). Complementation of the *Δppk2* mutant restored survival to levels comparable to WT (Figure 3B and C).

The role of PPK2 in *C. jejuni* aerotolerance was assayed by monitoring the survival of the *Δppk2* mutant under aerobic condition. Interestingly, the *Δppk2* mutant had a significant (*P*≤0.01) survival defect at 12 and 24 h compared to WT (Figure 3D). At 12 h, 3.2-log fewer bacteria were recovered from the *Δppk2* mutant compared to WT. At 24 h, no bacteria were recovered from the *Δppk2* mutant, while the WT showed 5×10^7^ CFU of bacteria. Complementation of the *Δppk2* mutant restored survival to levels comparable to WT (Figure 3D). Unlike the *Δppk2* mutant, the *ppk1* mutant has no survival defect under aerobic conditions [15], [16].

### The *Δppk2* mutant exhibits reduced capacity to form Viable But Non-culturable (VBNC) cells

**Figure 4:**
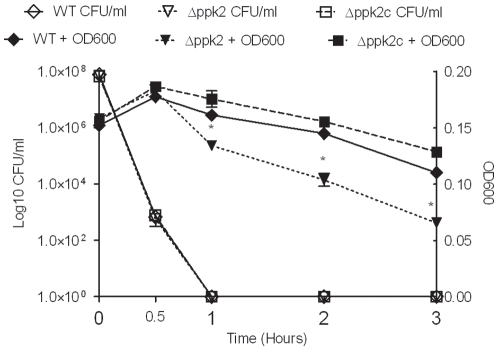
Culturability and viability of the *C. jejuni* strains after formic acid treatment. Culturability was determined by plating formic acid treated bacteria on MH agar at different time points. Viability was assessed by measuring OD_600_ after CTC staining. Each data point represents the mean±SE of 3 independent experiments. * *P*≤0.05.

VBNC formation is an important mechanism used by *C. jejuni* to survive in the environment under different stress conditions [29], [30]. The *Δppk2* mutant showed reduced VBNC formation under formic acid stress compared to WT (Figure 4). Though no culturable bacteria were recovered from the WT and *Δppk2* mutant at 1 h post-treatment, both strains retained viability until 3 h after formic acid treatment as determined by CTC staining. However, the *Δppk2* mutant showed significantly (*P*≤0.05) reduced viability at 1, 2 and 3 h post-treatment compared to WT and complementation of the Δ*ppk2* mutant restored the viability to levels comparable to WT (Figure 4).

### The *Δppk2* mutant exhibits enhanced biofilm formation

**Figure 5:**
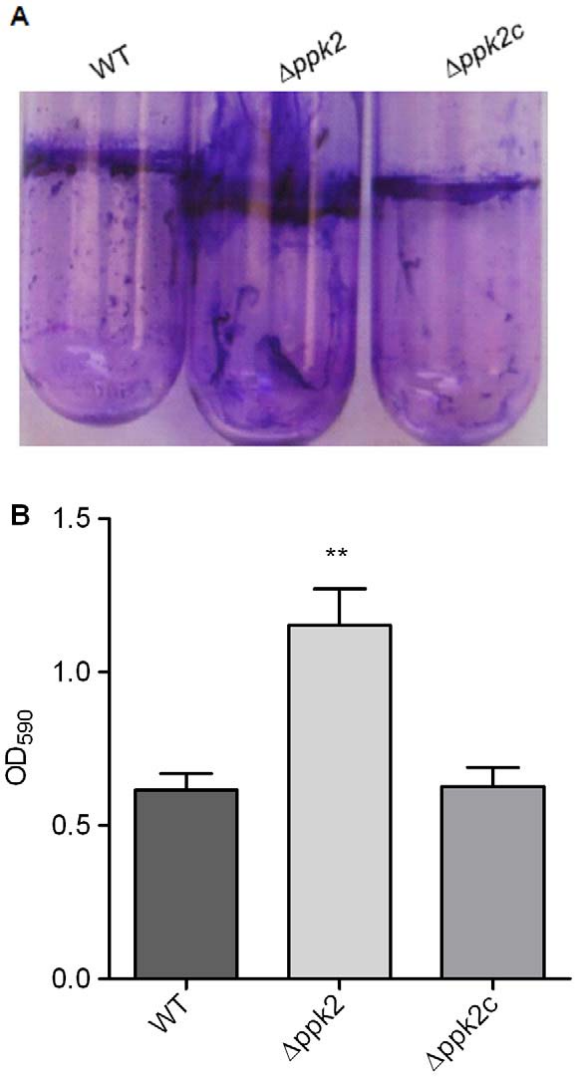
Biofilm formation in the *Δppk2* mutant. (**A**) The *Δppk2* mutant shows enhanced biofilm formation. Biofilm formation as assessed by crystal violet staining. (**B**) Quantification of biofilm using DMSO. The amount of biofilm formed was dissolved in 1 ml DMSO for 48 h and quantified by measuring absorbance at 570 nm. Each bar represents the mean±SE of 3 independent experiments. ** *P*≤0.01.

The *C. jejuni Δppk2* mutant had no motility defect when tested on semisolid agar (Table S1). However, when grown in MH broth microaerobically at 37°C, the *Δppk2* mutant formed more unattached aggregates at the bottom of the tube which suggested that the *Δppk2* mutant may display enhanced biofilm formation [31]. Indeed, the *Δppk2* mutant exhibited significantly (*P*≤0.01) enhanced biofilm formation compared to WT after 48 h of static growth (Figure 5A and B). Complementation of the *Δppk2* mutant decreased biofilm formation similar to WT levels (Figure 5, A and B).

### The *Δppk2* mutant shows increased susceptibility to antimicrobials

Since poly P plays a role in mediating antimicrobial resistance in *C. jejuni* [16], we asked whether *C. jejuni* PPK2 has a role in antimicrobial resistance. Though not significant, the *Δppk2* mutant exhibited increased susceptibility to several antimicrobials compared to WT (Table 1). Specifically, the *Δppk2* mutant showed increased susceptibility to both macrolides (erythromycin; 4-fold) and flouroquinolones (ciprofloxacin; 2-fold) that are considered drugs of choice for treating *Campylobacter* infections in humans. A low MIC was observed for tetracycline in the WT. The WT strain used in this study is cured for pTet plasmid that carries *tet(O)* gene which confers tetracycline resistance to *C. jejuni* [32]. This was further confirmed by PCR using *tet(O)* specific primers (data not shown). No difference in susceptibility was observed for other antibacterials and detergents including arsenical compounds (Table 1). Complementation of the *Δppk2* mutant restored the susceptibility to levels comparable to WT (Table 1).

**Table 1:** Susceptibility of the *Δppk2* mutant to various antimicrobials, bile acids and arsenical compounds.

Antimicrobial	WT	*Δppk2*	*Δppk2c*
		**MIC (µg/ml)**	
Azithromycin	0.12	0.06 (2)^a^	0.12
Ciprofloxacin	0.25	0.12 (2)	0.25
Erythromycin	0.5	0.125 (4)	0.5
Tetracycline	2	0.5 (4)	1.0
Fluorfenicol	2	0.5 (4)	1.0
Nalidixic acid	16	4 (4)	16
Telithromycin	2	1 (2)	2
Clindamycin	0.5	0.25 (2)	0.5
Gentamicin	0.5	0.5 (-)	0.5
Cefotaxime	1.6	1.6 (-)	1.6
Rifampin	100	100 (-)	100
Polymyxin B	3	3 (-)	3
Ethidium bromide	0.625	0.625 (-)	0.625
Cholic acid	6250	6250 (-)	6250
Taurocholic acid	36,000	36,000 (-)	36,000
Deoxycholic acid	16,500	16,500 (-)	16,500
Arsenite	64	64 (-)	64
Arsenate	1024	1024 (-)	1024
Roxarsone	128	128 (-)	128

^a^Fold difference between the WT and *Δppk2* mutant.

### The expression of *spoT*, *phosR*, *pstS*, *pstC*, *ppk1*, *csrA* and *cmeC* was up-regulated in the *Δppk2* mutant

**Figure 6:**
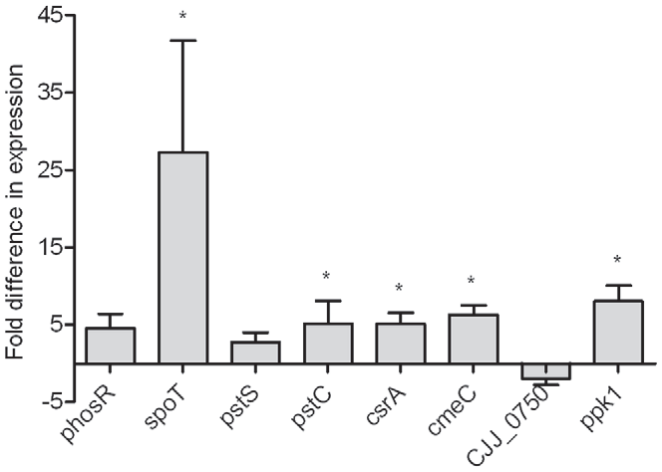
Quantitative RT-PCR analysis of the WT and *Δppk2* mutant. Fold differences in transcript levels were assessed from ΔΔCT after normalization using *rpoA*. Each bar represents the mean±SE of relative fold change in expression from 3 independent experiments. * *P*≤0.05.

To understand the mechanisms underlying PPK2-mediated phenotypes, we performed quantitative RT-PCR targeting genes encoding SpoT, a mediator of stringent response; PhosR, PstS and PstC which are involved in phosphate uptake; PPK1, a key protein in poly P synthesis; CsrA, a post-transcriptional regulator and CmeC, a component of multidrug efflux pump. The aforementioned genes were up-regulated (2-fold or more) in the *Δppk2* mutant compared to WT (Figure 6). However, only *spoT, ppk1, cmeC, pstC* and *csrA* showed significant up-regulation (*P*≤0.05). The transcription of genes involved in oxidative stress resistance [CJJ81176_0356 and, CJJ81176_0298 (anti-oxidant AhpCTSA family proteins)] and *sodB* was unaltered in the *Δppk2* mutant compared to WT (data not shown) suggesting that PPK2 is only involved in regulation of a subset of genes.

### The *Δppk2* mutant is defective in invasion and intracellular survival in INT407 cells

**Figure 7:**
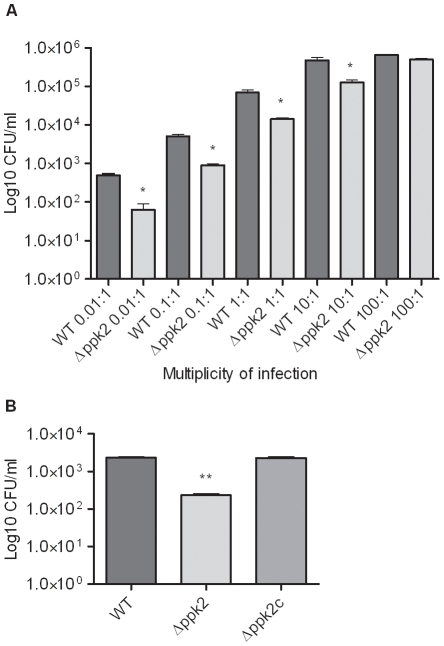
Invasion and intracellular survival of the *Δppk2* mutant in INT407 cells. (**A**) The *Δppk2* mutant displays a dose-dependent invasion defect in INT407 human intestinal epithelial cells. The data represents the average of 2 experiments with 3 replicates in each experiment. * *P*≤0.05. (**B**) The *Δppk2* mutant is defective in intracellular survival in INT407 cells. INT407 cells were infected with 100∶1 MOI of bacteria. Intracellular survival of *C. jejuni* was determined after 24 h of incubation. The data represents the average of 2 independent experiments with 3 replicates in each experiment. ** *P*≤0.01.

To investigate if PPK2 is involved in virulence-associated phenotypes, we examined whether the *Δppk2* mutant could adhere, invade and survive within INT407 human intestinal epithelial cells. Though the *Δppk2* mutant did not show a defect in adherence (data not shown), the mutant exhibited a significant (*P*≤0.05) dose-dependent defect in invasion (Figure 7A). Specifically, defect in invasion was more evident at a lower MOI (0.01∶1, 0.1∶1 and 1∶1). The *Δppk2* mutant also exhibited a significant (*P*≤0.01) defect in intracellular survival in INT 407 cells, while complementation restored the defect to WT values (Figure 7B).

### The *Δppk2* mutant exhibited a dose-dependent chicken colonization defect

**Figure 8:**
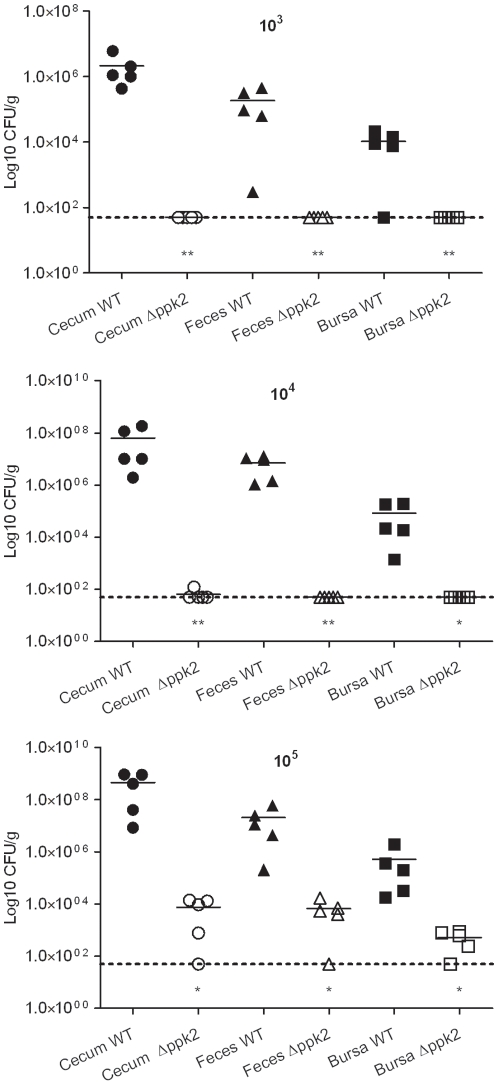
Colonization of the *Δppk2* mutant in chickens. The *Δppk2* mutant exhibits a dose-dependent colonization defect in day-old chicks. Eight days after inoculation, the chicks were sacrificed; cecum, feces and bursa were harvested and colonization level was assessed by determining the CFU/g of tissue. Each data point represents log_10_ CFU/g of tissue. Average CFU for each dose is denoted by a line. The dotted line indicates minimum detection limit of 50 CFU. * *P*≤0.05 and ** *P*≤0.01.

To test the contribution of PPK2 to *C. jejuni* host colonization, we tested the ability of the *Δppk2* mutant to colonize day-old chicks. The *Δppk2* mutant exhibited a significant (*P*≤0.01 or 0.05) dose-dependent colonization defect compared to WT (Figure 8). At 10^3^, 10^4^, and 10^5^ inoculation doses, the *Δppk2* mutant had significantly (*P*≤0.01 or 0.05) fewer average CFU in the cecal contents, feces and bursa compared to WT. At 10^3^ and 10^4^ inoculation levels, no detectable bacteria were recovered from the *Δppk2* mutant strain in all chicks from all organs tested except from cecal contents in one chicken at 10^4^ inoculation dose, while the WT strain colonized all organs in all chicks. These findings indicate that *Δppk2* mutant exhibits a significant colonization defect at lower levels of inoculum.

## Discussion

In the absence of classical stress response mechanisms which are crucial for the success of many foodborne pathogens, it is imperative for *C. jejuni* to use alternative mechanisms to survive in different environments. Poly P plays an important role in mediating bacterial survival under various stresses and stringencies [9], [12]. Here, we characterized the role of *ppk2* which is involved in poly P-dependent GTP synthesis. Our data suggest that *ppk2* might play a role in several phenotypes that are important for *C. jejuni* transmission, colonization, and persistence within and outside the host environment. Specifically, the Δ*ppk2* mutant was sensitive to aerobic, osmotic, nutrient, and antimicrobial stresses (Figure 3 and Table 1) as well as defective in chicken colonization (Figure 8).

The *C. jejuni* 81-176 *ppk2* is conserved among various *C. jejuni* strains and *Campylobacter* species with a sequence similarity ranging from 98–100% and 69–90%, respectively (data not shown). Based on the BLAST analysis and size agreement with the PPK2 homologs in other bacteria [27] including other *C. jejuni* strains, which all harbored the larger approximately 293 aa protein, ORFs 0632 (162 aa) and 0633 (137 aa) together seem to encode PPK2 in 81–176. However, further analysis is needed to identify whether 0632 and 0633 encode unique proteins in 81–176.

The *C. jejuni* PPK2 is a 1-domain protein and has high sequence similarity to *P. aeruginosa* PPK2 paralogs, PA0141 (61%), PA2428 (56%), and PA3455 (35%); *M. smegmatis* PPK2, SMEG_0891 (55%) and *M. tuberculosis* PPK2, rv3232c (48%) (Figure S2 and S3). The predicted tertiary structure of *C. jejuni* PPK2 sequence revealed a higher similarity to the C-terminal domain of *P. aeruginosa* PPK2 paralog PA3455 (Figure S4B). PPK2 class of enzymes belongs to the large superfamily of P loop kinases, which catalyze the hydrolysis or binding of nucleoside triphosphates [33]. The hallmark of P loop kinases is the presence of Walker A (GXXXXGK), and Walker B (hhhhD, where h is a hydrophobic residue) motifs as well as a lid module (Rx(2–3)R). The Walker A, and Walker B and the lid module in *C. jejuni* PPK2 are highly conserved (Figure S3 and S4A) as well as nine conserved residues within the catalytic site that are required for PPK2 activity in *P. aeruginosa* [27] are also present in *C. jejuni* PPK2 (Figure S3) suggesting that this protein has a similar catalytic mechanism.

The *P. aeruginosa* PPK2 (PA0141) and *M. tuberculosis* PPK2 (rv3232c) preferentially catalyze the synthesis of GTP using poly P as phosphate donor [22], [23]. The PA0141 also synthesizes poly P from GTP but at a 75-times lower rate than GTP synthesis from poly P, while rv3232c does not contribute to poly P synthesis. Contrary to PA0141 and rv3232c, *P. aeruginosa* PPK2 paralogs PA2428 and PA3455 primarily catalyze the synthesis of ATP/ADP from poly P and do not mediate poly P synthesis from ATP/GTP [27]. Our results showed that the *C. jejuni Δppk2* mutant was significantly defective in poly P-dependent GTP generation, indicating that *C. jejuni* PPK2 catalyzes poly P-driven GTP synthesis (Figure 1A) similar to PA0141 and rv3232c. However, the *C. jejuni Δppk2* mutant showed no defect in poly P-dependent ATP synthesis, rather it displayed increased ability to generate ATP resulting in increased intracellular ATP:GTP ratio (Figure 1B), suggesting that PPK2 plays a role in regulating intracellular nucleotide pool in *C. jejuni*. The Mycobacterial rv3232c and SMEG_0891 modify NDK, which is involved in the transfer of phosphate from ATP to other NTPs with preference to GTP synthesis [23]. In the *Mycobacterium ppk2* mutant, NDK lacked preferential synthesis of GTP suggesting that PPK2 modulates NDK activity. Interestingly, *C. jejuni* genome possesses a homolog of NDK with 48 and 47% sequence similarity to rv3232c and SMEG_0891, respectively. Thus, the possibility of a similar interaction of PPK2 to direct NDK towards GTP synthesis cannot be ruled out in *C. jejuni*.

The deletion of *ppk2* significantly reduced the *C. jejuni* survivability under nutrient, osmotic and aerobic stresses (Figure 3). *C. jejuni* responds to environmental stresses by eliciting a stringent response mediated by ppGpp, a signal molecule that regulates virulence and stringent response in *C. jejuni* and other bacteria [34]. GTP is a precursor for ppGpp synthesis [28], surprisingly, though not statistically significant, the *C. jejuni Δppk2* mutant showed slightly elevated ppGpp levels compared to WT (Figure 2A). NDK, in the absence of PPK2, might serve as an alternative source of GTP required for ppGpp synthesis as NDK can still contribute to residual levels of GTP. In addition, ppGpp regulates poly P levels in *E. coli* and high levels of ppGpp resulted in accumulation of large amounts of poly P as a result of inhibition of PPX, an enzyme involved in poly P degradation [35]. As expected, our findings showed no altered levels of poly P in the *Δppk2* mutant compared to WT (Figure 2B), possibly a consequence of no change in ppGpp levels. Consistent with our finding, a recent study in *M. smegmatis* also showed that deletion of *ppk2* did not alter intracellular poly P levels compared to WT [23]. However, *spoT* was up-regulated in our *C. jejuni Δppk2* mutant when grown in rich media (Figure 6). Unlike in other bacteria, SpoT is critical for both ppGpp synthesis as well as its hydrolysis in *C. jejuni* [34]. In nutrient-rich conditions, SpoT hydrolyzes ppGpp to GTP and inorganic phosphate, keeping ppGpp levels low and preventing the cells from entering stress response and growth arrest [36]. Thus, the up-regulation of *spoT* in the *C. jejuni Δppk2* mutant may be a compensatory response to keep ppGpp levels low preventing cells from entering stress response. Despite unaltered intracellular ppGpp and poly P levels, it is intriguing to note that the *C. jejuni Δppk2* mutant showed significant defect in stress tolerance and pathogenicity, suggesting that these phenotypes probably are not mediated by a ppGpp-mediated stringent response. Alternatively, some of the phenotypes in the *C. jejuni Δppk2* mutant may be due to the potential role of PPK2 in regulating other virulence and stress response-related genes [9], [24], [25], [37].

Adherence, invasion and intracellular survival are important virulence mechanisms in *C.* *jejuni* pathogenesis [38], [39] as these attributes permit evasion of immune response, in addition to their role in cellular damage, relapse and persistence in the human host [40], [41], [42]. The *C. jejuni Δppk2* mutant was significantly defective in invasion (Figure 7A) and intracellular survival (Figure 7B) within human intestinal epithelial cells, suggesting an important role for PPK2 in *C. jejuni* pathogenesis. Our observation that the Δ*ppk2* mutant is sensitive to a variety of *in vitro* stresses is consistent with the fact that *C. jejuni* must overcome nutrient and acid stresses within the intestinal epithelial cells, providing an explanation for the intracellular survival defect in the *Δppk2* mutant.

Our study describes for the first time the importance of PPK2 *in vivo* in bacteria. The *C. jejuni Δppk2* mutant displayed a significant dose-dependent colonization defect in chickens (Figure 8). Inactivation of *ppk1* which encodes a poly P synthesizing enzyme [15], [16] and *cprS* which encodes a sensor kinase [43] in *C. jejuni* also resulted in a dose-dependent colonization defect in chickens. An enhancement in biofilm formation was proposed as the possible explanation for dose-dependent chicken colonization defect in *ppk1* and *cprS* mutants. Specifically, at lower doses, the mutant may be significantly susceptible to *in vivo* stresses; however, at higher doses, a dose-dependent hyperbiofilm phenotype was suggested to restore resistance of the mutant to *in vivo* stresses similar to WT. A similar mechanism might explain the dose dependent colonization defect in *C. jejuni Δppk2* mutant as the *Δppk2* mutant also exhibited hyperbiofilm phenotype similar to *ppk1* and *cprS* mutants (Figure 5, A and B). However, it should be noted that the defect in chicken colonization by the *Δppk2* mutant is multifactorial.

Similar to the *ppk1* mutant, resistance to oxidative, heavy metal, heat, anaerobic, and acid (acetic, propionic, and hydrochloric acids) stresses were not affected in the Δ*ppk2* mutant (Table S1). The Δ*ppk2* mutant was also not defective in iron utilization and growth at non-permissive temperatures (4 and 25°C) (Table S1). Although *C. jejuni* PPK2 is structurally not related to PPK1, certain phenotypes are common to both PPK1 and PPK2, while others are not, suggesting that PPK1 and PPK2 contribute to overlapping but not redundant functions. Most importantly, the absence of either PPK1 or PPK2 compromises *C. jejuni* physiology and pathogenesis. Although PPK1 and PPK2 might work by distinct mechanisms, PPK1 may require direct or indirect participation of PPK2 for particular functions and vice-versa, which partially explains the overlapping phenotypes in these proteins. Our repeated efforts to generate a *ppk1* and *ppk2* double mutant to better understand the role of these genes in *C. jejuni* patho-physiology were unsuccessful, suggesting that either *ppk1* or *ppk2* is required for *C. jejuni* viability. Consistent with our finding, a recent report in *M. smegmatis* also showed that viable double knockouts lacking both *ppk1* and *ppk2* could not be obtained [23]. Though, the exact mechanism by which poly P, PPK1 and PPK2 mediate stress response and virulence in *C. jejuni* is unknown, this study adds to our understanding of the contribution of poly P-associated proteins in *C. jejuni* adaptation and survival under different stringencies in the absence of RpoS-mediated classical stress response mechanisms.

## Materials and Methods

### Ethics statement

Animal experiment was conducted according to the guidelines of Association for Assessment and Accreditation of Laboratory Animal Care International (AAALAC). The animal studies are approved by Agricultural Animal Care and Use Committee (AgACUC), OARDC, The Ohio State University under the protocol number 07-AG007. By necessity, microbial pathogenesis studies are heavily focused on the use of *in vivo* models as the host immune responses interplay with pathogen evasion, a complex response that cannot yet be accurately replicated *in vitro*. Chicks are the natural source of human infections and chick colonization model is widely used for *Campylobacter* studies. Many of the virulence factors that are relevant to human pathogenesis have also been shown to be important for chick colonization. We designed our experiments to use the minimal number of animals required to generate reliable data. As we study complex host pathogen interactions that are applicable to human health, the use of laboratory animals is unavoidable and justifiable.

Chickens were housed at the Food Animal Health Research Program Animal Care Facility. The facility is fully accredited by AAALAC. Infectious agents were administered using manual restraint for less than one minute to minimize distress. Chickens were euthanized by carbon dioxide inhalation, which is rapid and painless. This method is consistent with the recommendations of the panel on euthanasia of the American Veterinary Medical Association and by The Ohio State University Institutional Laboratory Animal Care and Use Committee.

### Bacterial strains, media and growth conditions

Bacterial strains and plasmids used in this study are listed in Table S2. *C. jejuni* strain 81–176 (WT), a highly invasive strain originally isolated from an outbreak associated with raw milk [44], was used to generate the *ppk2* deletion mutant. *C. jejuni* strains were routinely grown on Mueller-Hinton broth (MH; Oxoid) microaerobically [(85% N_2_ (v/v), 10% CO_2_ (v/v) and 5% O_2_ (v/v)] in a DG250 Microaerophilic Workstation (Microbiology International) at 42°C. MH agar plates were supplemented with *Campylobacter* selective supplement (SR117E, Oxoid) when isolating *C. jejuni* from chicken feces and organs. For growth curve and stress survival assays, *C. jejuni* was grown microaerobically in MH broth with appropriate antibiotics at 37°C with shaking at 200 rpm. *E. coli* DH5α was used for plasmid propagation and cloning purposes and was routinely cultured on Luria-Bertani (LB) medium at 37°C overnight. Growth media was supplemented with appropriate antibiotics, chloramphenicol (20 µg/ml for *E. coli*; 10 µg/ml for *Campylobacter*), kanamycin (30 µg/ml) and zeocin (50 µg/ml), where necessary.

### General cloning techniques

Cloning and other molecular biology techniques were performed according to Sambrook and Russel [45]. Oligonucleotides were designed using Vector NTI® software (Invitrogen) and commercially synthesized by Integrated DNA Technologies. All the oligonucleotides used in the present study are listed in Table S3. Masterpure® DNA purification kit and Fast-Link DNA ligation kit were purchased from Epicentre. Restriction enzymes were purchased from Promega. QIAquick® PCR purification kit and QIAprep® spin mini prep kit for plasmid isolation were purchased from Qiagen. Zero background cloning vector pZErO-1 and *E. coli* DH5α competent cells were purchased from Invitrogen.

### Targeted deletion of *ppk2* in *C. jejuni*

BLAST search using *ppk2* sequence from other *C. jejuni* strains showed that *C. jejuni* 81–176 harbored CJJ81176_0632 (encoding 162 aa) and CJJ81176_0633 (137 aa) which together had a 98–100% sequence similarity with Cj0604 (NCTC 11168), C8J_0566 (81116) and CJE0707 (RM 1221), each of which encode a putative PPK2 with 293 aa in length. The CJJ81176_0633 is annotated as *ppk2* while CJJ81176_0632 is annotated as a hypothetical protein in the NCBI genome database. Based on the BLAST analysis and size agreement with the PPK2 homologs in other bacteria [27] including other *C. jejuni* strains, which all harbored the larger approximately 293 aa protein, these two genes seem to encode PPK2 in 81–176. Consequently, in this study, we deleted most of the coding sequence (95%) of both CJJ81176_0632 and 0633 together in the same mutant to ensure that the PPK2 function is completely abolished.

Deletion of *ppk2* (0632–0633) was achieved by double crossover homologous recombination using a suicide vector containing approximately 1 kb of homologous sequences on either side of *ppk2* gene as described previously [16]. Briefly, *ppk2* along with 1 kb flanking region on either side of the target gene was amplified by PCR using PPK2 F and PPK2 R primers from *C. jejuni* 81–176 genomic DNA. The amplified PCR product was ligated into pZErO-1 to generate plasmid pDG4. Inverse PCR was performed on pDG4 using PPK2 INV F and PPK2 INV R primers to delete majority of the *ppk2* coding sequence. Kanamycin cassette from pUC4K was then cloned into inverse PCR product, the resulting suicide vector designated pDG5 was electroporated into *C. jejuni* 81–176 as described [46]. Recombinants were selected on MH agar plates containing kanamycin, kanamycin resistant colonies were streak purified and one such mutant designated DG003/Δ*ppk2* was used for further studies. The deletion of the *ppk2* gene was confirmed by PCR.

### Complementation of the *Δppk2* mutant

The *ppk2* coding sequence (0632–0633 included) along with the potential promoter region was amplified by PCR using PPK2 COMP F and PPK2 COMP R primers. The amplified PCR product was ligated to pRY111, an *E. coli*-*Campylobacter* shuttle vector [47], and the resulting complementation plasmid pDG6 was introduced into the *Δppk2* mutant by triparental conjugation as described previously [48]. Transconjugants were selected on MH agar plate containing kanamycin and chloramphenicol and one such transconjugant designated DG004*/Δppk2*c was used in complementation studies to confirm the specific effects of *ppk2* deletion except for invasion, intracellular survival and chicken colonization studies in which case it is difficult to maintain the selective pressure.

### Growth curve assay

Mid-log phase grown cultures of *C. jejuni* were diluted to an OD_600_ of 0.05 in MH broth and incubated microaerobically for 60 h at 42°C with shaking at 200 rpm. For assessing the growth and culturability, serial 10-fold dilutions of the cultures at different time points were plated on MH agar and CFU were determined.

### Growth kinetics after pyruvate kinase inactivation

Growth kinetics of *C. jejuni* after inactivation of pyruvate kinase by Tween 20 was performed as described [25]. Briefly, mid-log phase grown cultures were diluted to an OD_600_ of 0.05 in MH broth containing 0.1% (v/v) Tween 20 and incubated microaerobically at 42°C for 60 h. At different time points, serial ten-fold dilutions of cultures were plated on MH agar and CFU were determined.

### Assay for PPK2 as a poly P-dependent GTP/ATP generator

Preparation of crude lysate for PPK2 activity was performed as described previously [22]. Briefly, mid-log phase grown cultures of *C. jejuni* were diluted to an OD_600_ of 0.05 in MH broth and incubated microaerobically for 16 h at 42°C with shaking at 200 rpm. Cells were harvested by centrifugation at 5,000× *g* for 5 min and resuspended in TED buffer (50 mM Tris HCl, pH 8.0/0.5 mM EDTA/1 mM DTT). Cells were sonicated and centrifuged at 10,000× *g* for 10 min and the crude lysate was assayed for PPK2 activity. Poly P-dependent synthesis of ATP/GTP was determined by using a modified enzyme-coupled assay with hexokinase and glucose-6-phosphate dehydrogenase [27]. Assay mixture (100 µl) contained 10 mM MnCl_2_ (for GTP)/MgCl_2_ (for ATP), 80 mM (NH_4_)_2_SO_4_, 50 mM Tris pH 8.0, 5 mM GDP/ADP, 1.5 mM NADP, 1 mM glucose, 3 U yeast hexokinase, 1.5 U *Leuconostoc mesenteroides* glucose-6-phosphate dehydrogenase, 1–2 mg crude lysate and 5 mM sodium polyphosphate (P_12–13_, added at last). Hexokinase converts GTP/ATP generated by PPK2 to GDP/ADP and glucose-6-phosphate using glucose as the phosphate acceptor and the glucose-6-phosphate will be converted to 6-phosphogluconate by NADP-dependent glucose-6-phosphate dehydrogenase. During this reaction, NADP will be converted to NADPH which was measured spectrophotometrically at 340 nm (ε340 nm = 6.22 mM^−1^ cm^−1^). NADPH formed was measured up to 10 min and the enzyme activity was expressed as Vmax as described before [27]. The enzyme kinetics was analyzed by nonlinear curve fitting using GraphPad Prism 5.0 software.

Further, the amount of GTP and ATP in the above PPK2 reaction products was calculated using TLC. Briefly, 10 µl of the reaction mixture, normalized by volume, was loaded on to TLC plate and run using saturated ammonium sulfate, 3 M sodium acetate and 2-Propanol (80∶6∶2, v/v/v) as a solvent system. Nucleotides were visualized using UV light, developed by ninhydrin and quantified by densitometry using Image J Software from NIH. Quantifications are presented as fold increase or decrease with respect to WT strain.

### ppGpp isolation and detection

ppGpp was assayed as described previously [34]. Briefly, the bacterial strains were grown to early exponential phase (OD_600_∼0.3). Cultures were diluted to OD_600_ of 0.1 in MH broth and incubated for 2 h. Following incubation, the OD_600_ was adjusted to ∼0.25; cells were pelleted, washed twice in MOPS-MGS (50 mM MOPS, 55 mM mannitol, 1 mM MgSO_4_, 0.25 mM CaCl_2_, 19 mM glutamic acid, and 0.004 mM biotin) [49] and resuspended in 250 µl MOPS-MGS. The ^32^P at 100 µCi ml^−1^ (3.7×10^12^ Bq) was added to cells and incubated for 1 and 3 h at 37°C microaerobically. Labeled cells were harvested, washed and treated with lysozyme in 10 mM Tris (pH 8.0) for 20 min. The cells were lysed using 1% SDS (w/v) and ppGpp was extracted with equal volume of 2 M formic acid and placed on ice for 15 min. Samples were spun for 5 min at 10,000× *g*, and 3 µl of supernatant was spotted directly onto cellulose TLC plates, dried, and developed in 1.5 M KH_2_PO_4_ and visualized by autoradiography.

### Quantification of poly P

Poly P was extracted using glassmilk and quantified using toluidine blue O as described earlier [15]. Poly P was quantified from mid-log, late-log and mid-stationary phase cultures by measuring the ratio of 530 to 630 nm spectrophotometrically using appropriate concentrations of phosphorous standard (Sigma).

### Survival under nutrient stress

To determine survival under nutrient stress, mid-log phase grown bacterial cultures were pelleted, washed twice with MEM and resuspended in MEM as described previously [15], [16]. The OD_600_ was adjusted to 0.05 and the cultures were incubated microaerobically at 42°C for 60 h with shaking at 200 rpm. One hundred microliters of culture at different time points was serially diluted (10-fold), plated on MH agar and CFU were determined.

### Osmotic stress survival

Osmotic stress tolerance was determined as described previously [15], [16]. To assess the osmotic stress survival in liquid culture, bacterial strains were grown to mid-log phase, adjusted to an OD_600_ of 0.05 in MH broth with and without 0.25 M NaCl and incubated microaerobically at 42°C for 60 h with shaking at 200 rpm. One hundred microliters of the culture at different time points was serially diluted (10-fold) in MH broth containing 0.25 M NaCl and plated on MH agar. The plates were incubated microaerobically and CFU were determined. To determine osmotic stress tolerance on solid media, mid log-phase grown WT, Δ*ppk2,* and *Δppk2c* cultures were serially diluted (10-fold), 10 µl of diluted culture was spotted on MH agar containing 0.17 M NaCl and incubated microaerobically at 42°C for 2 days.

### Aerobic survival

Aerobic survival of *C. jejuni* was assessed by exposing the bacteria to aerobic conditions and determining their culturability at different time points. Briefly, *C. jejuni* was grown to mid-log phase and adjusted to an OD_600_ of 0.05. The samples were incubated at 42°C under aerobic conditions with shaking for 60 h and 100 µl of ten fold serial dilutions of the culture was plated on MH agar at different time points and CFU were determined.

### Induction and enumeration of VBNC cells in *C. jejuni*

VBNC cells in *C. jejuni* were induced as described previously [16], [50]. Briefly, 1 ml of overnight grown culture containing 5×10^8^ CFU was added to 4 ml of MH broth with pH adjusted to 4.0 using formic acid and incubated microaerobically at 42°C for 3 h. VBNC formation was confirmed by determining the total culturable counts and viability. Total culturable counts were determined by plating the formic acid treated bacteria at different times for enumerating CFU. Viability was determined by measuring OD_600_ after CTC staining [16]. Briefly, the bacterial samples were stained by adding 5-cyano-2, 3-ditolyl tetrazolium chloride (CTC: Polysciences) to a final concentration of 5 mM for 1 h at room temperature in dark. Cells were pelleted, pellet was resuspended in 500 µl of PBS and viability was determined by measuring CTC reduction spectrophotometrically at 600 nm.

### Biofilm formation

Static biofilm formation was assessed in borosilicate tubes as described previously [15], [16], [51] by inoculating 100 µl of 0.05 OD_600_ culture into MH broth and incubating at 42°C microaerobically for 2 days without shaking. Biofilms were visualized by staining with 250 µl of 1% (w/v) crystal violet for 15 min, and quantified by measuring the absorbance at 570 nm after dissolving in 1 ml DMSO for 48 h.

### Antimicrobial susceptibility testing

The susceptibility of the WT, *Δppk2* and *Δppk2c* strains to different antimicrobials was determined by microtiter broth dilution using polypropylene plates as described previously [16], [52]. Briefly, 100 µl of mid-log phase grown cultures adjusted to an OD_600_ of 0.05 in MH broth was added to microtitre plate containing serially (2-fold) diluted antimicrobials. Plates were incubated at 42°C microaerobically at 200 rpm for 2 days without shaking, and minimal inhibitory concentration (MIC, µg/ml) was determined by recording the lowest concentration of an antimicrobial showing complete inhibition of visible bacterial growth.

Susceptibility to azithromycin, ciprofloxacin, erythromycin, tetracycline, florfenicol, nalidixic acid, telithromycin, clindamycin, and gentamicin was determined by using Sensititre® susceptibility plates for *Campylobacter* (TREK Diagnostic). Briefly, one hundred microliters of log-phase grown cultures adjusted to an OD_600_ of 0.05 in MH broth was added to each well in the Sensititre® susceptibility plate and the wells were covered using the perforated adhesive seal. Plates were incubated microaerobically at 42°C for 24 h and MIC was recorded. Results were read following the manufacturer's instructions and interpreted according to MIC interpretive guidelines by Clinical Laboratory Standards Institute. The susceptibility testing was repeated 3 times and mean MIC was calculated.

### Quantitative RT-PCR

Quantitative RT-PCR (qPCR) was performed targeting key genes involved in phosphate uptake (*phosR*, *pstS*, *pstC*, and periplasmic substrate binding protein, CJJ81176_0750) [53], [54], [55], stringent response (*spoT*) [34], and multidrug resistance (*cmeC*) [52], post transcriptional global regulator (*csrA*) [56] and poly P synthesis (*ppk1*) [15], [16]. Total RNA was extracted from log-phase grown bacterial cultures using RNeasy Mini Kit (Qiagen). The RNA concentration and purity was determined using NanoDrop ND-1000 spectrophotometer. cDNA synthesis was carried out using SuperScript® III First-Strand Synthesis SuperMix (Invitrogen). Gene specific primers were designed to amplify the abovementioned genes along with *rpoA* (internal control) using Beacon Designer 7.0. The *rpoA* has recently been shown to be the most suitable internal control for qRT-PCR analysis of stress responses and growth phase effects in *C. jejuni* [57]. qPCR was performed using SensiMixPlus® SYBR RT-PCR Kit (Quantace) in a realplex^2^ mastercycler (Eppendorf). The relative levels of expression of genes were normalized with *rpoA* amplified from the corresponding sample. The difference in expression of the genes was calculated using the comparative threshold cycle (CT) method to yield fold-difference in transcript levels.

### Adherence, invasion and intracellular survival in INT407 cells

Adherence, invasion and intracellular survival assays were performed as described previously [34], [43]. Each well of a 24-well tissue culture plate was seeded with 1.4×10^5^ INT 407 cells in MEM with 10% (v/v) fetal bovine serum (FBS) and incubated for 18 h at 37°C with 5% CO_2_. *C. jejuni* strains were grown to mid-log phase in MH broth microaerobically, the cells were pelleted at 5,000× *g* for 10 min, washed twice with MEM containing 1% (v/v) FBS, resuspended in MEM to an OD_600_ of 0.02 and used for infection. INT407 cells were infected with different multiplicities of infection (MOI), 100∶1 for adherence and intracellular survival assays and 0.01∶1, 0.1∶1. 1∶1, 10∶1 and 100∶1 for invasion assay. For infection, 1 ml of bacterial cell suspension was pipetted on to INT 407 cells, centrifuged at 1000× *g* for 3 min and incubated for 3 h. For determining adherence, cells were rinsed with MEM three times, lysed with 0.1% (v/v) Triton-X 100 and diluted serially in MEM and plated on MH agar in duplicate to determine CFU. For determining invasion, after 3 h of incubation with bacteria, cells were treated with gentamicin (150 µg/ml) and incubated for additional 2 h. After 2 h of incubation, the infected cells were rinsed with MEM three times, lysed with 0.1% (v/v) Triton-X 100, serially diluted in MEM and plated on MH agar in duplicate to determine CFU. To assess intracellular survival, following 2 h gentamicin treatment, the infected cells were washed with MEM three times and covered with MEM containing gentamicin (10 µg/ml) and incubated for 24 h. After 24 h of incubation, the infected cells were washed with MEM, lysed with 0.1% (v/v) Triton-X 100, serially diluted in MEM and plated on MH agar in duplicate to determine CFU.

### Chicken colonization studies

Chicken colonization studies were performed as described previously [15], [16]. Colonization experiments were conducted according to the guidelines of AAALAC. Briefly, day-old broiler chicks (n = 5 for each group) from a local hatching facility (Food Animal Health Research Program, OARDC, Wooster, OH) were inoculated orally with 10^3^, 10^4^ and 10^5^ CFU of the *C. jejuni* WT and *Δppk2* mutant strains in 200 µl of PBS (pH 7.4). Eight days post-inoculation, the chicks were euthanized, cecal contents, feces and bursa were collected aseptically, weighed, homogenized, diluted in PBS (pH 7.4) and plated on MH agar containing *Campylobacter* selective supplement. Plates were incubated at 42°C microaerobically and CFU per gram of tissues were determined.

### Statistical analysis

Statistical significance of data generated in this study was determined using one-way analysis of variance (ANOVA) followed by Tukey's HSD (Honestly Significant Difference) test or Student's t-test (paired 2-tailed). *P*≤0.01 or 0.05 (α level) was considered statistically significant.

## Supporting Information

Table S1 Phenotypes with no significant difference between the WT and the Δppk2 mutant.
(0.08 MB DOC)

Table S2 Bacterial strains and plasmids used in this study.
(0.06 MB DOC)

Table S3 Primers used in this study.
(0.06 MB DOC)

Figure S1 Growth kinetics of the C. jejuni 81–176 WT and Δppk2 mutant assessed by CFU determination. Each data point represents the mean ± SE of 3 independent experiments.
(0.12 MB PPT)

Figure S2 Phylogram of PPK2 from C. jejuni and its near neighbors. Branch lengths are indicated next to the protein name and are proportional to the predicted evolutionary change. Phylogram was constructed using ClustalW2.
(0.27 MB PPT)

Figure S3 Structure-based sequence alignment of PPK2 domains from C. jejuni. P. aeruginosa, M. tuberculosis and M. smegmatis. Strictly conserved residues are highlighted by white letters on grey background. The conserved motifs Walker A and Walker B are indicated by triangles and squares, respectively. Lid module is indicated by dashed line. *indicates residues critical for PPK2 catalysis. PA0141 and PA2428-P. aeruginosa 1-domain PPK2 paralogs; PA3455-C- C-terminal domain of P. aeruginosa 2-domain PPK2 paralog PA3455; PA3455-N- N-terminal domain of P. aeruginosa 2-domain PPK2 paralog PA3455; CJJ81176_0632/633-C. jejuni PPK2; rv3232c-M. tuberculosis PPK2 and SMEG_0891-M. smegmatis PPK2. Sequence alignment was performed using ClustalW2 (www.ebi.ac.uk/Tools/clustalw2/index.html).
(0.06 MB DOC)

Figure S4 Structure of PPK2. (A) Predicted three-dimensional structure of C. jejuni PPK2. Three-dimensional structure was identified with vector alignment search tool (www.ncbi.nlm.nih.gov/Structure/VAST/vast.shtml) using P. aeruginosa PPK2 paralog PA3455 as reference. Walker A, Walker B and lid module are indicated by letters A, B and C in yellow, respectively. The region in pink or red indicates C. jejuni PPK2 residues identical to PA3455. The region in grey indicates unaligned sequences of C. jejuni. (B) C. jejuni PPK2 superimposed on P. aeruginosa PPK2 paralog PA3455. Note that PA3455 has 4 domains (PA3455 is a 2-domain PPK2 and exists as a dimer). C and N indicate C- and N-terminal domains. C. jejuni PPK2 superimposes only with the C-terminal domain of PA3455. Superimposed structures were obtained using VAST and Cn3D structure and sequence alignment viewer.
(0.69 MB PPT)
