# Supplementary material for: Polyphosphate Kinase 2: A Novel Determinant of Stress Responses and Pathogenesis in Campylobacter jejuni
Source: PLoS One. 2010 Aug 17;5(8):e12142. doi: 10.1371/journal.pone.0012142 (PMC2923150; doi:10.1371/journal.pone.0012142)
Supplement: Table S1 — Phenotypes with no significant difference between the WT and the Δppk2 mutant. (0.08 MB DOC) [file pone.0012142.s001.doc]

**Table S1**. Phenotypes with no significant difference between the WT and the *∆ppk2* mutant.

| **Phenotypic assay** | **Method** | **WT** |  | ***∆ppk2*** |
| --- | --- | --- | --- | --- |
| **Motility** | Semisolid agar (0.4 %) stab assay | 28 mm |  | 26 mm |
| **Acid stress**  **Acetic acid**  **Propionic acid**  **HCl** | CFU determination of acid exposed bacteria (pH 4.0 in MH broth) at different time points |  | No difference  No difference  No difference |  |
| **Heat shock at 55° C for 1, 2, 3 and 5 min** | CFU determination |  | No difference |  |
| **Anaerobic survival** | CFU determination after anaerobic growth using BBL Gaspak Plus Anaerobic system |  | No difference |  |
| **Survival at non-growth temperatures**  **4° C**  **25° C** | CFU determination |  | No difference  No difference |  |
| **Growth difference between 37 and 42° C** | CFU determination |  | No difference |  |
| **Growth after pyruvate kinase inactivation** | CFU determination |  | No difference |  |
| **Oxidative stress**  **H_2_O_2_**  **0.03 %**  **0.1 %**  **0.3 %**  **Paraquat**  **1 mM**  **10 mM** | Disc (6 mm) diffusion assay | 3 mm  7.5 mm  12 mm  3 mm  16 mm |  | 3 mm  7 mm  11.5 mm  3 mm  16 mm |
| **Heavy metal tolerance**  **Arsenite**  **Arsenate**  **Roxarsone**  **CuSO4 (50 mM)**  **ZnSO4 (50 mM)** | MIC determination by microdilution method  (µg/ml)  Disc (6 mm) diffusion assay | 64 µg  1024 µg  128 µg  5 mm  4.5 mm |  | 64 µg  1024 µg  128 µg  4.5 mm  4.5 mm |
| **Iron chelation**  **Desferal (0.05 mM)**  **Dipyridyl (0.05 mM)** | Disc (6 mm) diffusion assay | 24 mm  27 mm |  | 22 mm  26 mm |
| **Chemotaxis for L-fucose** | By determining the zone of attraction on 0.4 % PBS soft agar |  | No difference |  |
| **Adherence to INT407 cells** | By CFU determination |  | No difference |  |
