# Supplementary material for: Polyphosphate Kinase 2: A Novel Determinant of Stress Responses and Pathogenesis in Campylobacter jejuni
Source: PLoS One. 2010 Aug 17;5(8):e12142. doi: 10.1371/journal.pone.0012142 (PMC2923150; doi:10.1371/journal.pone.0012142)
Supplement: Table S2 — Bacterial strains and plasmids used in this study. (0.06 MB DOC) [file pone.0012142.s002.doc]

**Table S2.** Bacterial strains and plasmids used in this study.

| **Strain/Plasmid** | **Relevant description** | **Source/Reference** |
| --- | --- | --- |
| ***C. jejuni* 81-176 WT** | WT strain of *Campylobacter jejuni* | Qijing Zhang |
| ***∆ppk2*/ DG003** | *C. jejuni* 81-176 derivative with deletion in *ppk2* gene; *ppk2*::Kan | This study |
| ***∆ppk2c*/ DG004** | DG003 harboring pDG6, Cm | This study |
| ***E. coli* DH5α** | *E. coli* strain used for cloning | Invitrogen |
| **pZERO-1** | Cloning vector for making suicide vector; Zeo | Invitrogen |
| **pRY111** | *E. coli*-*Campylobacter* shuttle vector for complementation | [58] |
| **pRK2013** | Helper plasmid for conjugation | [12] |
| **pUC4K** | Source for kanamycin | Amersham |
| **pDG4** | pZErO-1 containing *ppk2* region plus 1 kb upstream and downstream sequences from 81-176; Zeo | This study |
| **pDG5** | Suicide vector with *ppk2* replaced by kanamycin resistance region from pUC4K in pDG4; Zeo, Kan | This study |
| **pDG6** | pRY111 containing *ppk2* coding region and the upstream promoter sequence for complementation; Cm | This study |

Kan, kanamycin; Cm, Chloramphenicol; Zeo, zeocin
