# Supplementary material for: Polyphosphate Kinase 2: A Novel Determinant of Stress Responses and Pathogenesis in Campylobacter jejuni
Source: PLoS One. 2010 Aug 17;5(8):e12142. doi: 10.1371/journal.pone.0012142 (PMC2923150; doi:10.1371/journal.pone.0012142)
Supplement: Table S3 — Primers used in this study. (0.06 MB DOC) [file pone.0012142.s003.doc]

**Table S3.** Primers used in this study.

| **Name** | **Sequence (5’-3’)** |
| --- | --- |
| **Primers used for construction of *∆ppk2* mutant and *∆ppk2c* complemented strains** | |
| PPK2 F  PPK2 R  PPK2 INV F  PPK2 INV R  PPK2 COMP F  PPK2 COMP R | ATAAAAGGTACCAGGTTTGAAATCTTAATGGC  AAAAAACTGCAGCAATGGAGGAAATTCTTTAG  ATAAAAGGATCCAAATTAGTGCGTAGTGGAGA  AAAAAAGGATCCAACCCTTGGCTTTAACATGA  TAATAACTGCAGACGCATAAATTTCCTAAAAG  ATATATGGTACCGCATTCTTTTCCTTAATCTA |
| **Primers used for quantitative RT-PCR** | |
| CsrA F  CsrA R  SpoT F  SpoT R  PhosR F  PhosR R  CmeC F  CmeC R  PstS F  PstS R  PstC F  PstC R  CJJ81176_0750 F  CJJ81176_0750 R  PPK2-RT F  PPK2-RT R  PPK1-RT F  PPK1-RT R  RpoA F  RpoA R  CJJ_0298 F  CJJ_0298 R  CJJ_0356 F  CJJ_0356 R  SodB F  SodB R | TTATCGGAGAAGGTATAG  TTTCTAAGTATCATAAGGG  GTAACCACTCGCACAATATC  GATGTCGCAGTTTATTCTCC  GCAAACATAATCATCACAACCAC  GAGAGCAAGGATACAAAGAAGC  GCTGCTGCTCAATTAGGTATAG  GCTTCATAATCATACTCACTTGC  CCTTATACAAACTGGAATCAAATC  GACACATCACTCATTACAAGC  CGCTTATGCTTTAGGTATGAC  GCTGCCATCACCACTATC  GGTCTTGTTGCCTTATTG  GTATCGCTATGTTCTATGC  ATCTAATACTCCAACTTGTC  TTCTTCTTCTCCACTACG  TGAAGCAAGTATGGAAGGAG  ATATAGGAGTCATAAGTTCTAAGC  ATTACAACATCTGCTTATACG  TCTACTATTTCTTTATTTGATTCG  GATTATTGGTATTAGTCCTGATAG  AAGTAGAACGAATGATGCC  AGTAATTGGAATTTCAGG  TAAATCATTAACCACAGC  TTATCAAAGGTGCTACAGGAG  CAAACATCTACAACAAGTAAAGG |
