# Supplementary figures and images for: Polyphosphate Kinase 2: A Novel Determinant of Stress Responses and Pathogenesis in Campylobacter jejuni
Source: PLoS One. 2010 Aug 17;5(8):e12142. doi: 10.1371/journal.pone.0012142 (PMC2923150; doi:10.1371/journal.pone.0012142)

## Slide 1
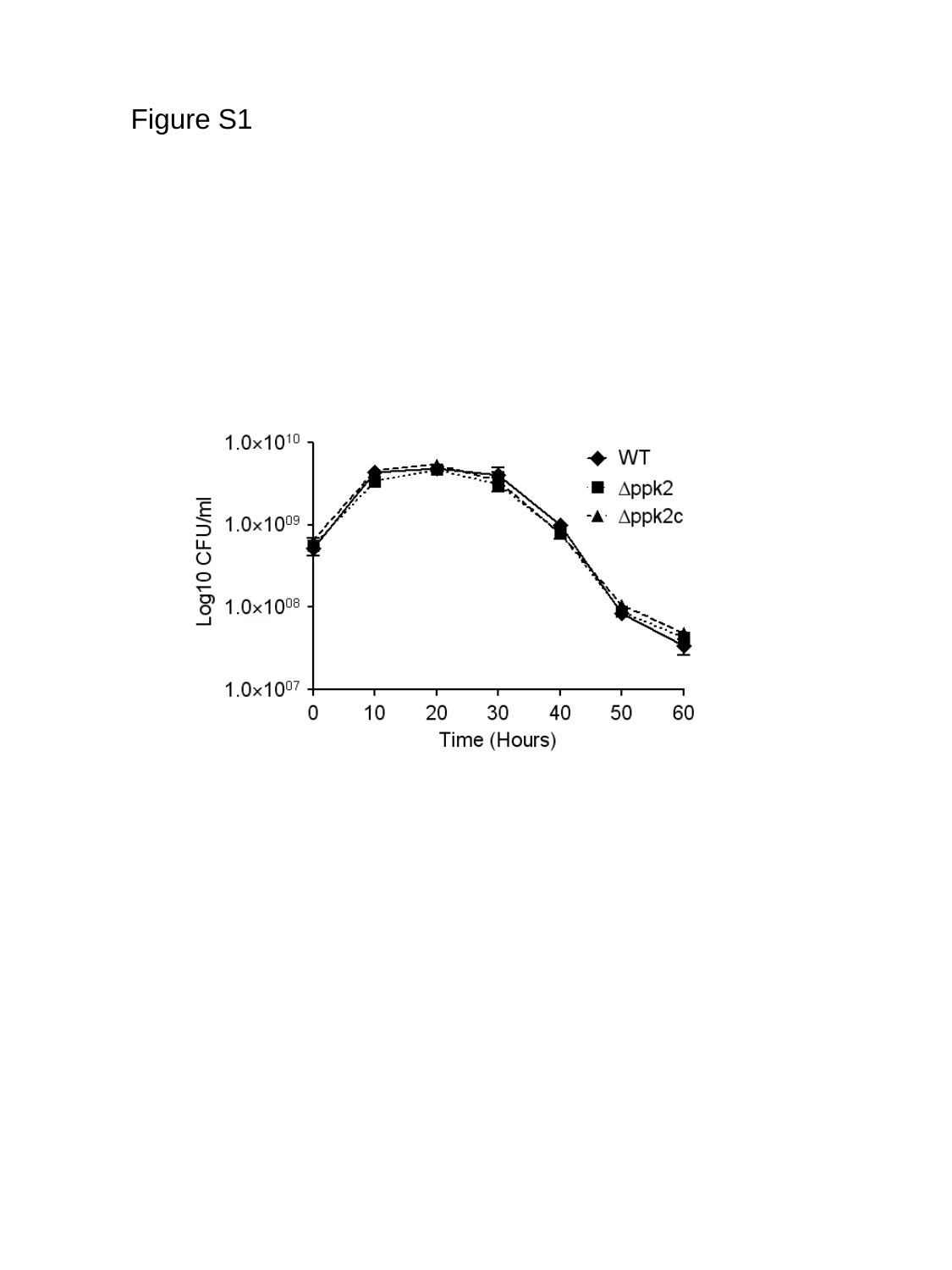

Figure S1

Supplement: Figure S1 — Growth kinetics of the C. jejuni 81–176 WT and Δppk2 mutant assessed by CFU determination. Each data point represents the mean ± SE of 3 independent experiments. (0.12 MB PPT) [file pone.0012142.s004.ppt]

## Slide 1
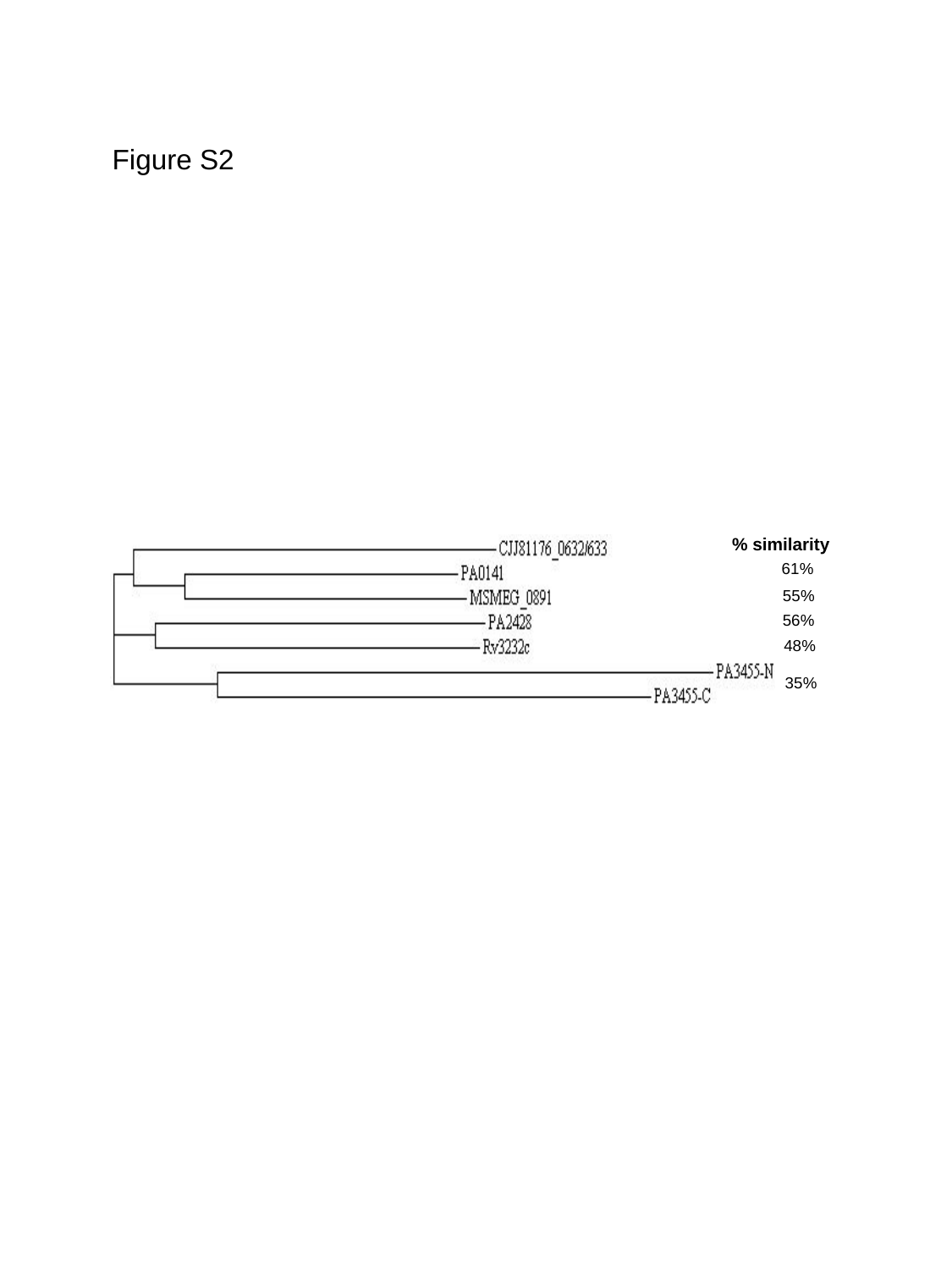

Figure S2
% similarity
61%
55%
56%
48%
35%

Supplement: Figure S2 — Phylogram of PPK2 from C. jejuni and its near neighbors. Branch lengths are indicated next to the protein name and are proportional to the predicted evolutionary change. Phylogram was constructed using ClustalW2. (0.27 MB PPT) [file pone.0012142.s005.ppt]

## Slide 1
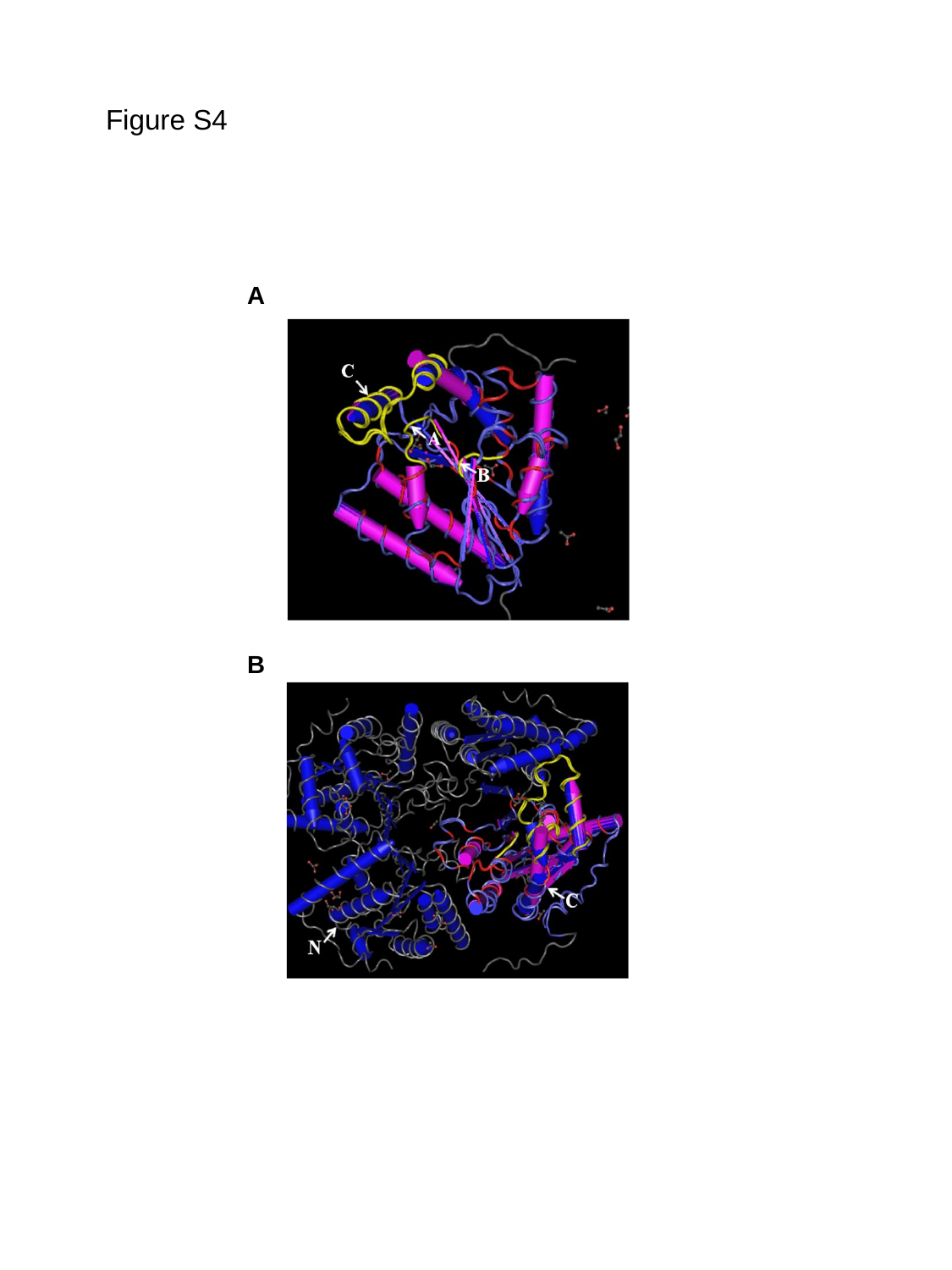

Figure S4
A
B

Supplement: Figure S4 — Structure of PPK2. (A) Predicted three-dimensional structure of C. jejuni PPK2. Three-dimensional structure was identified with vector alignment search tool (www.ncbi.nlm.nih.gov/Structure/VAST/vast.shtml) using P. aeruginosa PPK2 paralog PA3455 as reference. Walker A, Walker B and lid module are indicated by letters A, B and C in yellow, respectively. The region in pink or red indicates C. jejuni PPK2 residues identical to PA3455. The region in grey indicates unaligned sequences of C. jejuni. (B) C. jejuni PPK2 superimposed on P. aeruginosa PPK2 paralog PA3455. Note that PA3455 has 4 domains (PA3455 is a 2-domain PPK2 and exists as a dimer). C and N indicate C- and N-terminal domains. C. jejuni PPK2 superimposes only with the C-terminal domain of PA3455. Superimposed structures were obtained using VAST and Cn3D structure and sequence alignment viewer. (0.69 MB PPT) [file pone.0012142.s007.ppt]
