# Supplementary material for: Polyphosphate Kinase 2: A Novel Determinant of Stress Responses and Pathogenesis in Campylobacter jejuni
Source: PLoS One. 2010 Aug 17;5(8):e12142. doi: 10.1371/journal.pone.0012142 (PMC2923150; doi:10.1371/journal.pone.0012142)
Supplement: Figure S3 — Structure-based sequence alignment of PPK2 domains from C. jejuni. P. aeruginosa, M. tuberculosis and M. smegmatis. Strictly conserved residues are highlighted by white letters on grey background. The conserved motifs Walker A and Walker B are indicated by triangles and squares, respectively. Lid module is indicated by dashed line. *indicates residues critical for PPK2 catalysis. PA0141 and PA2428-P. aeruginosa 1-domain PPK2 paralogs; PA3455-C- C-terminal domain of P. aeruginosa 2-domain PPK2 paralog PA3455; PA3455-N- N-terminal domain of P. aeruginosa 2-domain PPK2 paralog PA3455; CJJ81176_0632/633-C. jejuni PPK2; rv3232c-M. tuberculosis PPK2 and SMEG_0891-M. smegmatis PPK2. Sequence alignment was performed using ClustalW2 (www.ebi.ac.uk/Tools/clustalw2/index.html). (0.06 MB DOC) [file pone.0012142.s006.doc]

**Figure S3**

PA0141 ---------------MALQVASAPHGSSEDSTSASLPAN---------YPYHTRMRRNEY 36

MSMEG_0891 ---------------MLDSTGYAVRDDDDDDPELLLPGGEVVDTWREGYPYDERMHRADY 45

CJJ81176_0632/633 ---------------MQENNSPKAQAVVKKN-EIYVSVK-------------RKKSTIEY 31

PA2428 MDSYGDTSGRIGRDWLDRHDEELEQELLDDELNLDELFGP----EQEDAPG--ELSRRRY 54

Rv3232c ---------------MDIPSVDVSTATNDGAS--SRAKG-----HRSAAPGRRKISDAVY 38

PA3455-C -------------AKRQPHAAPLVSSLDNRGLLDSLDLG-------------QYLDKDAY 34

PA3455-N ----------------MFESAEVGHS----------------------------IDKDTY 16

* * *

▲▲▲▲▲▲▲

PA0141 EKAKHDLQIELLKVQSWVKETG-QRVVVLFEGRDAAGKGGTIKRFMEHLNPRGARIVALE 95

MSMEG_0891 EEQKRLLQIELLKLQKWSQAHG-HRHVIVFEGRDAAGKGGTIKRFMEHLNPRGARVVALE 104

CJJ81176_0632/633 EKDLKNLQIELLKFQNHVKAKG-LKVLILIEGRDAAGKGGAIKRLIEHLNPRGCRVVALE 90

PA2428 FRELFRLQRELVKLQNWVVHTG-HKVVILFEGRDAAGKGGVIKRITQRLNPRVCRVAALP 113

Rv3232c QAELFRLQTEFVKLQEWARHSG-ARLVVIFEGRDGAGKGGAIKRITEYLNPRVARIAALP 97

PA3455-C KEQLAAEQARLAGLIRDKRFRQ-HSLVAVFEGNDAAGKGGAIRRVTDALDPRQYHIVPIA 93

PA3455-N EKAVIELREALLEAQFELKQQARFPVIILINGIEGAGKGETVKLLNEWMDPRLIEVQSFL 76

**

■■■

PA0141 KPSSQEQGQWYFQRYIQHLPTAGEMVFFDRSWYNRAGVERVMGFCSPLQYLEFMRQAPEL 155

MSMEG_0891 KPTERERTQWYFQRYVEHLPAAGELVLFDRSWYNRAGVERVMGYCTPKQHAEFIRQAPLF 164

CJJ81176_0632/633 KPSDVEKTQWYFQRYIAHLPSAGEIVIFDRSWYNRAGVEPVMGFCTPQQHKDFLREVPLF 150

PA2428 APNDREQTQWYFQRYVSHLPAGGEIVLFDRSWYNRAGVERVMGFCNDEQYEEFFRSVPEF 173

Rv3232c APTDRERGQWYYQRYIAHLPAKGEIVLFDRSWYNRAGVEKVMGFCTPQEYVLFLRQTPIF 157

PA3455-C APTEEERAQPYLWRFWRHIPARRQFTIFDRSWYGRVLVERIEGFCAPADWLRAYGEINDF 153

PA3455-N RPSDEELERPPQWRFWRRLPPKGRTGIFFGNWYSQMLYARVEGHIKEAKLDQAIDAAERF 136

* * * *

-------------------------------

PA0141 ERMLTNSGILLFKYWFSVSREEQLRRFISRRDDPLKHWKLSPIDIKSLDKWDDYTAAKQA 215

MSMEG_0891 EQMLVNDGISLTKLWFSVTRSEQLTRFTIRQVDPVRQWKLSPTDLASLDKWDDYTAAKEE 224

CJJ81176_0632/633 ENMISNSDIIFFKFYFSVSKDEQKKRFEKRRSDPLKQYKLSPVDQKSQELWDKYTLAKYS 210

PA2428 EKMLARSGIQLLKYWFSISDAEQHLRFLSRIHDPLKQWKLSPMDLESRRRWEAYTKAKET 233

Rv3232c EQMLIDDGILLRKYWFSVSDAEQLRRFKARRNDPVRQWKLSPMDLESVYRWEDYSRAKDE 217

PA3455-C EEQLSEYGIIVVKFWLAIDKQTQMERFKEREKTPYKRYKITEEDWRNRDKWDQYVDAVGD 213

PA3455-N ERMLCDEGALLFKFWFHLSKKQLKERLKALEKDPQHSWKLSPLDWKQSEVYDRFVHYGER 196

PA0141 MFFHTDTADAPWTVIKSDDKKRARLNCIRHFLHSLDYPDKDRRIAHEPDPLLVGPASRVI 275

MSMEG_0891 MFAWTDTEIAPWTVVKSNDKKRARINAMRYVLGKFDYDNKDHEVVGQADPLIVG---RAL 281

CJJ81176_0632/633 MLLASNTPTCPWTIISSDDKKKARLNLLRFILSKVEYPNKKTGDFSKIDAKLVRSGEEEI 270

PA2428 MLERTHIPEAPWWVVQADDKKRARLNCIHHLLQQMPYREVPQPPVHLPER-LRHADYVRH 292

Rv3232c MMVHTDTPVSPWYVVESDIKKHARLNMMAHLLSTIDYADVEKPKVKLPPRPLVSGNYRRP 277

PA3455-C MVDRTSTEIAPWTLVEANDKRFARVKVLRTINDAIEAAYKKDK----------------- 256

PA3455-N VLRRTSRDYAPWYVVEGADERYRALTVGRILLEGLQAALATKER---------------- 240

PA0141 EEDEKVYAEAAAAPGHANLDIPA 298

MSMEG_0891 SD--------------------- 283

CJJ81176_0632/633 RKMEANLEKLDSKKADEKIKDLD 293

PA2428 PTPGEIIVPEVY----------- 304

Rv3232c PRELSTYVDDYVATLIAR----- 295

PA3455-C -----------------------

PA3455-N -----------------------
